# Social Engagement and Depressive Symptoms Among Middle-Aged and Older Adults in India

**DOI:** 10.1093/geroni/igab046.815

**Published:** 2021-12-17

**Authors:** Kasturi Banerjee, Tamara Baker

**Affiliations:** 1 University of Kansas, Lawrence, Kansas, United States; 2 University of North Carolina, Chapel Hill School of Medicine, University of North Carolina at Chapel Hill (School of Medicine), North Carolina, United States

## Abstract

Social networks and family support are known to have benefits for many older adults globally, and India is no exception. However, the exact nature of the impact of these factors on depression in the Indian population remains largely unexplored. Considering the aging Indian population and changing socio-cultural landscape, it is important to identify the role of social engagement and neighborhood factors in the mental health needs of these older adults. To address this need, this study aimed to identify predictors of depressive symptoms among individuals aged 45+ years from four states in India. Data were taken from the Longitudinal Aging Study in India (LASI) pilot survey in 2010, focusing on the influence social activities, family factors and neighborhood satisfaction variables have on depressive symptomatology. A hierarchical multiple regression analysis was conducted and found that residing in a southern state (Karnataka and Kerala) (β=.178, p<0.05); lower life satisfaction (β= -.261, p<0.05); having more living children (β=.110, p<0.05), less frequently or never visiting friends and relatives (β=.079, p<0.05) and not liking the neighborhood (β=.072, p<0.05) were predictors of depressive symptoms. These findings are consistent with limited extant literature on the importance of family and social engagement as predictors of depressive symptoms in this population. Future research should focus on qualitatively examining the interaction between depressive symptoms and social engagement within this population, thereby helping develop targeted interventions, measure outcomes and long term, community engagement-based prevention programs.

